# Effect of Palletisation and Temperature on Bag-in-Box Wine Packaging Under Simulated Export Conditions

**DOI:** 10.3390/foods15173138

**Published:** 2026-09-04

**Authors:** Nicola Mercanti, Monica Macaluso, Bruno Augusto Casu De Sousa, Kamran Mehravar, Fabrizio Palla, Piero Giorgio Verdini, Angela Zinnai

**Affiliations:** 1 Department of Agriculture, Food and Environment, University of Pisa, Via del Borghetto 80, 56124 Pisa, Italy; nicola.mercanti@phd.unipi.it (N.M.); angela.zinnai@unipi.it (A.Z.); 2 INFN Pisa Section, Largo Bruno Pontecorvo 3, 56127 Pisa, Italy; bruno.casu@cern.ch (B.A.C.D.S.); k.mehravar@studenti.unipi.it (K.M.); fabrizio.palla@cern.ch (F.P.); piero.giorgio.verdini@cern.ch (P.G.V.); 3 European Organization for Nuclear Research, Espl. des Particules 1, 1211 Meyrin, Switzerland; 4 Interdepartmental Research Centre “Nutraceuticals and Food for Health”, University of Pisa, Via del Borghetto 80, 56124 Pisa, Italy

**Keywords:** wine logistics, pressure variation, storage stability, sensor-based monitoring

## Abstract

Bag-in-box (BiB) wine packaging is increasingly used for export, but conditions inside palletised units are rarely measured directly. We instrumented 3 L BiB units with in-bag MEMS pressure and temperature sensors during a 20-day static chamber trial using 19 °C and 50 °C set-point chambers, labelled C19 and C50. The set-point labels do not represent measured wine temperatures. A 15-day single-stack verification trial was also run at the C50 set point. In the main trial, the measured in-bag temperature differed strongly from the chamber set point. C50 packages stabilised at 34.2 ± 0.4 °C in the top units and 26.1 ± 0.2 °C in the bottom units, while C19 units remained around 22.8–23.5 °C. In the verification trial, individual sensors reached 45.0–48.8 °C, but no in-bag sensor reached 50 °C. Internal pressure rose after handling and peaked between day 3 + 13 h and day 5 + 19 h. Bottom-position units showed a larger uncorrected peak ΔP than top-position units (adjusted difference: 20.4 mbar; F(1,8) = 9.14; *p* = 0.017). The position result is interpreted as exploratory. The pressure analysis used a small unbalanced sensor-level design, with one missing bottom-position C19 trace, no barometric reference logger, and no retained stack pairings. Bulk wine from C50 showed lower free and total SO_2_ and higher volatile acidity than wine from C19. Lactic acid was significantly higher in C50, although the underlying mechanism could not be established and residual microbial activity cannot be excluded. Overall, chamber set points were a poor proxy for actual in-package conditions. Selected sentinel packages can document real thermal and pressure exposure in palletised BiB wine.

## 1. Introduction

Wine packaging is increasingly evaluated not only for product protection but also for environmental and logistical performance. Glass remains the dominant format, but export requirements, material use, and waste management have encouraged interest in lighter alternatives [1,2,3,4,5].

The bag-in-box (BiB) format is one such alternative. It combines a corrugated cardboard outer case with a flexible inner bag and dispensing valve, uses less packaging mass per litre than glass, and is available in formats from about 3 to 20 L [6,7,8,9,10,11,12,13,14]. Its lower transport weight can reduce distribution impacts, but the same flexibility that gives BiB practical advantages also makes its mechanical response under stacking load important.

During international transport, wine packages may experience temperature fluctuations, vibration, vertical compression, and pressure changes for several weeks [15,16]. In BiB packaging, the flexible wall can deform and the headspace can compress or exchange gas through the film. The cardboard case also carries part of the pallet load.

Previous studies have examined storage temperature and packaging effects on wine composition, including sulphur dioxide loss, volatile acidity, and sensory ageing [17,18,19]. Most of that evidence concerns bottled wines, static storage, or chemical composition alone. Less is known about the pressure inside palletised BiB units during static chamber storage or about how measured in-package thermal exposure compares with chamber set points.

This study tested whether stack position and a severe chamber set point affected internal pressure and selected chemical parameters of commercial 3 L BiB white wine during static chamber storage. The two main-trial chambers are labelled C19 and C50 according to their 19 °C and 50 °C set points. These labels refer to chamber settings, not measured wine temperatures. The original intention was to compare a reference condition with a high-temperature condition. Because the main trial did not achieve 50 °C in-bag exposure, the realised comparison is between set-point groups and measured package temperatures, with support from a single-stack verification trial at the C50 set point.

## 2. Materials and Methods

The study investigated selected storage stresses that wine packages may encounter during export logistics under static chamber conditions. It did not reproduce vibration, humidity, diurnal thermal cycling, or a full container thermal profile. The main trial lasted 20 days. Palletised units were stored in parallel in two temperature-controlled chambers labelled by set point:

• C19: 19 °C chamber set point, used as the reference condition.
• C50: 50 °C chamber set point, selected as a deliberately severe set point relevant to worst-case non-refrigerated storage scenarios rather than routine shipment temperature [15].
• The labels C19 and C50 refer only to chamber set points; measured in-bag temperatures are reported separately. A 15-day verification trial was also carried out at the C50 set point with a single three-box stack (Section 2.3). The two trials are summarised in Table 1.

**Figure 1:**
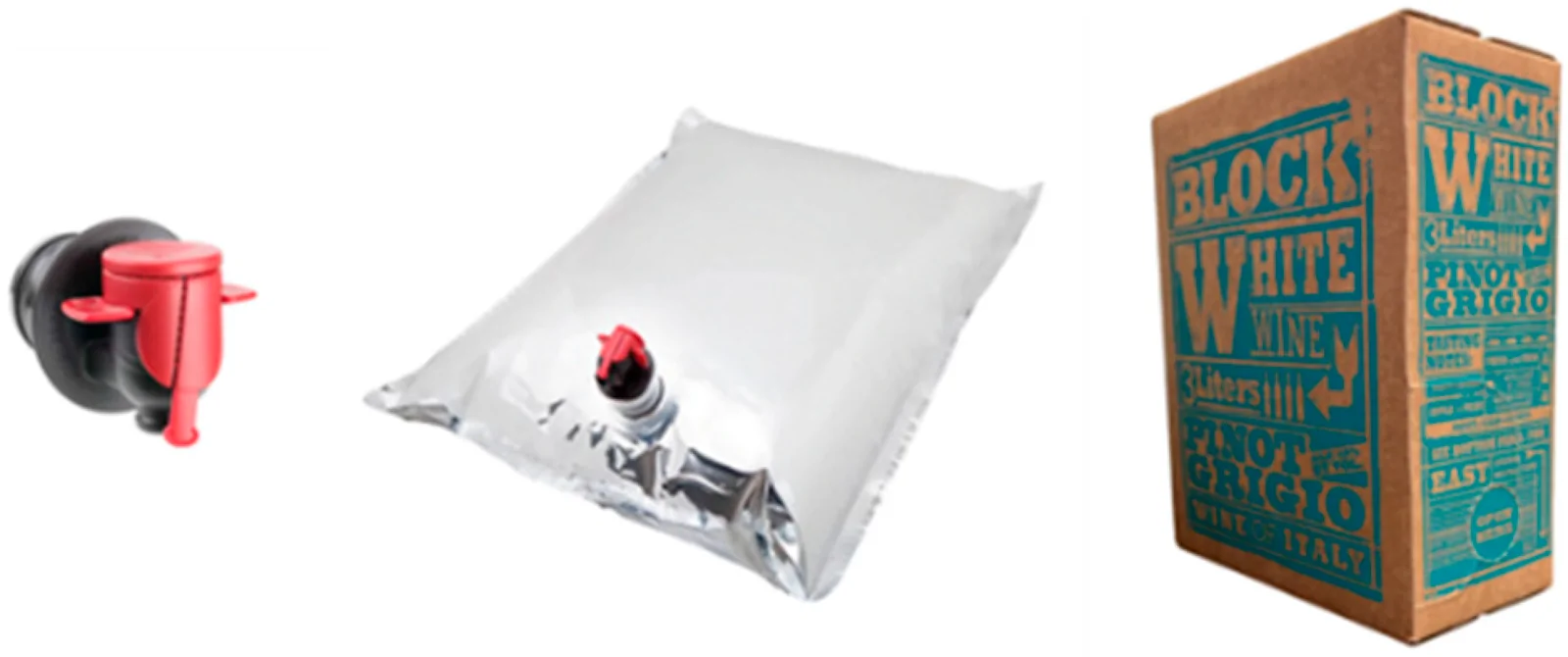
Principal components of bag-in-box (BiB) packaging system: dispensing tap, flexible multilayer wine bag with valve, and external corrugated cardboard container used for mechanical protection and pallet stability.

The wine used in this study was commercial white wine supplied by Castellani S.p.A. (Pontedera, Italy) and packaged in standard 3 L bag-in-box units representative of those used for commercial export (Figure 1). The supplier did not provide grape variety or vintage information. The main compositional parameters measured after the reference condition are reported in Table 2. The inner bag consisted of a flexible multilayer film with an oxygen-barrier layer and an integrated dispensing valve, housed in a corrugated cardboard case that provided mechanical protection during palletisation.

**Figure 2:**
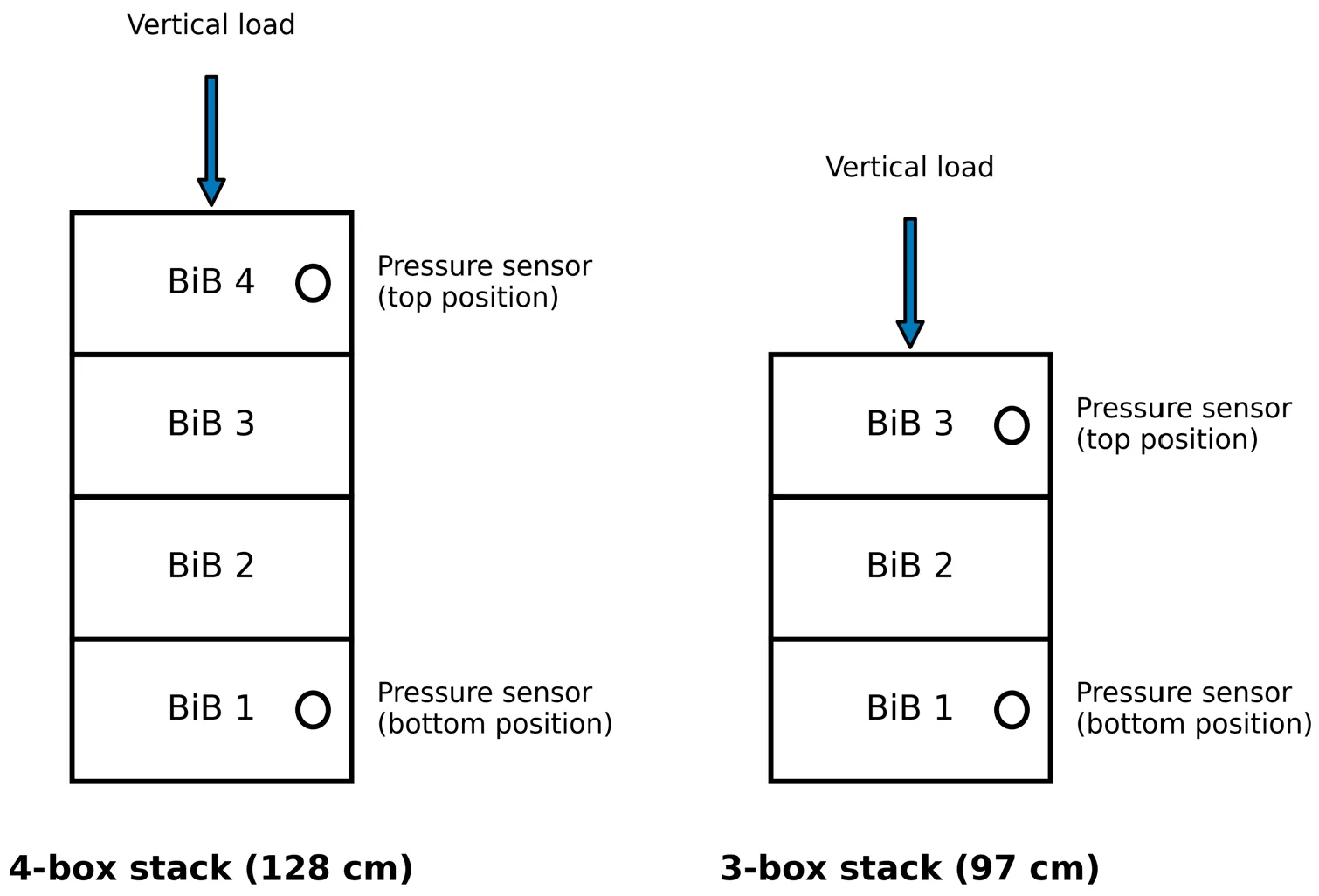
Experimental configuration used for pressure monitoring in palletised 3 L bag-in-box (BiB) units. Main trial included one 4-box stack (128 cm) and two 3-box stacks (97 cm) in each set-point chamber, C19 and C50. MEMS pressure sensors were positioned in uppermost and lowermost BiBs of each stack to compare package position under tested stacking configuration. Stack height was not analysed as independent factor because only one 4-box stack was present per chamber.

For each set-point group, three stacks were tested simultaneously: one stack of four boxes (128 cm) and two stacks of three boxes (97 cm) per chamber (Figure 2; Table 1). MEMS pressure sensors were positioned in the uppermost and lowermost BiB of every stack, giving six intended traces per chamber and twelve in total. One bottom-position sensor in C19 did not return a complete record, leaving eleven usable pressure traces. The pressure comparison therefore had cells of *n* = 3, 3, 3, and 2. The top and bottom observations came from different BiBs within the same stacks, so they represent instrumented package positions within stacks rather than repeated measurements of the same bag. The 20-day window was chosen to approximate the duration of transoceanic shipment, which is commonly within two to four weeks [15].

The records did not include the chamber model, volume, fan power, airflow rate, or formal pallet occupancy. These missing details limit the interpretation of the thermal mismatch and vertical temperature gradient in the main trial.

### 2.1. Chemical Parameters

At the end of the thermal exposure period, chemical checks were carried out to assess selected compositional differences in wine from C19 and C50.

The measured chemical variables were free SO_2_, total SO_2_, titratable acidity (g/L tartaric acid), volatile acidity (g/L acetic acid), ethanol, malic acid, lactic acid, total phenols, and pH. They were determined according to protocols described previously [20]. The reported values are determined in triplicate for the main-trial wine grouped by set-point label. Chemical sampling was not resolved by stack position. No microbiological measurements or viable cell counts were performed, although such data would be needed to evaluate residual fermentation activity [21]. Raw replicate-level chemical records were not retained in the available study records.

### 2.2. Physical Parameters

Pressure monitoring within the bag-in-box containers was carried out using custom-designed sensing units, as described in a previous study [22]. Each unit was built around an MS5803-05BA miniature altimeter module (TE Connectivity, Schaffhausen, Switzerland) and a 24-bit MEMS pressure and temperature sensor with SPI and I2C interfaces. According to its data sheet [23], the device covers 0–5 bar and −40 to +85 °C. It resolves pressure to 0.036–0.195 mbar, depending on the oversampling ratio, and temperature to better than 0.01 °C. The specified pressure accuracy is ±1.5 mbar between 300 and 1100 mbar at 20 °C, widening to ±100 mbar over the full 0–5 bar and 0–50 °C envelope. Long-term pressure stability is specified as ±1 mbar per year, and temperature accuracy as ±2.5 °C. The sensor was driven by an ESP8285 microcontroller on an ESP-M2 board (Adafruit, New York, NY, USA) [24] and powered by a lithium battery. The assembly was enclosed in an oil-filled plastic casing. The oil transmitted wine pressure to the transducer and dispersed the small amount of heat released by the electronics, whose draw is below 1 mA. The casing was introduced into each bag through the existing filling/dispensing valve before final sealing. The multilayer film was therefore not punctured. Sealing then followed the standard filling-line procedure, which was intended to minimise leakage and artificial perturbation of internal pressure.

Twelve sensing units were deployed in the main trial, with two per stack (uppermost and lowermost BiB) across the three stacks of each chamber. Pressure and temperature were recorded every 30 min throughout storage. Each pressure trace was referred to its own initial pressure and analysed as pressure change (ΔP), so the comparison depends on changes within each sensor rather than on agreement between different sensors. Because there was one chamber per set point, the chamber condition and chamber identity cannot be separated. The chamber factor is therefore interpreted as a set-point comparison, not as a replicated test of actual wine temperature.

### 2.3. Verification Trial at the C50 Set Point

The verification trial was carried out to document package behaviour closer to the intended high-temperature exposure. A single stack of three boxes of the same 3 L BiB format was instrumented with four MEMS sensors of the same type and held for 15 days at the C50 set point. V26 and V62 were installed in the uppermost BiB; V27 and V64 were installed in the lowermost BiB. Pressure and temperature were logged every minute, compared with the 30 min interval used in the main trial. V26 stopped after about 4 days and 8 h, and the remaining three sensors completed the record. The trial provides supporting observations for this configuration only.

### 2.4. Statistical Analyses

A one-way analysis of variance was performed using CoStat, Version 6.451, CoHort 6.0 software (Pacific Grove, CA, USA) to assess differences in the compositional parameters among the investigated samples. Means were separated by Tukey’s post hoc test at *p* ≤ 0.05. The pressure data were analysed separately because each instrumented BiB was described by stack position (top or bottom) and set-point group (C19 or C50). For each sensor, two summary responses were extracted from the ΔP curve: the post-handling peak within the 20-day storage period and the residual value on day 20. Pressure change was referenced to each sensor’s initial valid pressure reading at time zero. The primary exploratory analysis used the following additive two-way linear model:

(1) *y_ijk_* = μ + α*_i_* + β*_j_* + (αβ)*_ij_* + ε*_ijk_*

in which y is the summary value from one instrumented BiB, μ is the overall mean, α is the position term, β is the set-point term, and ε is the residual. The position-by-set-point interaction was checked separately with the full model and was not statistically significant, so the additive model is reported. The model assumes independent residuals with constant variance. With only eleven usable traces, these assumptions cannot be tested with high power. The top and bottom observations are package-position measurements within stacks, and the chamber factor is unreplicated at the chamber level.

(2) F = MS_factor_/MS_error_ = (SS_factor_/df_factor_)/(SS_error_/df_error_)

With two levels per factor and eleven usable traces, the additive model leaves one degree of freedom for each factor and eight residual degrees of freedom, giving the notation F(1,8). The design is unbalanced because one bottom-position sensor in C19 returned no complete record; the four cells therefore contain three, three, three, and two traces. Sums of squares were computed by the Type II method, in which each main effect is adjusted for the other [25]. Sensor-level *p*-values are reported within this exploratory analysis. Lack of significance is interpreted only as lack of a statistically detectable differences in this small design. Because stack identifiers and sensor-to-stack pairings were not retained, the *p*-values should not be read as definitive stack-level tests. The computation was carried out in Python 3 using the commented script, raw-workbook extraction path, and derived per-sensor summaries prepared for the pressure ANOVA [26].

## 3. Results and Discussion

### 3.1. Chemical Parameters

The chemical comparison used bulk wine from the main trial grouped as C19 or C50 (Table 2). These labels denote chamber set points, not wine temperatures. The chemical results therefore describe differences between set-point groups, not the effect of exposing wine to 50 °C.

Lactic acid was significantly higher in C50 wine (0.29 ± 0.02 g/L) than in C19 wine (0.16 ± 0.05 g/L) (Table 2). However, the available measurements do not allow the mechanism underlying this difference to be established. Malic acid did not differ significantly between C50 and C19 (1.0 ± 0.16 and 1.1 ± 0.23 g/L, respectively), but the absence of a statistically significant decrease in malic acid is not sufficient to exclude limited malolactic activity. Since no microbiological measurements or viable cell counts were performed, residual malolactic or other microbial activity cannot be ruled out [21].

Volatile acidity, expressed as acetic acid, was higher in C50 wine (0.39 ± 0.03 g/L) than in C19 wine (0.30 ± 0.02 g/L) (Table 2). This value is below the commonly used spoilage limits for table wine, but the study did not include sensory testing. The difference is consistent with warmer storage effects, including the oxidative and hydrolytic pathways described for wine storage [18,19]. A microbiological contribution cannot be excluded because microbiological measurements were not collected.

The largest practical between-condition difference concerned total and free SO_2_, both of which were lower in C50 wine. Total SO_2_ was 25 ± 8.2 mg/L in C50 compared with 50 ± 5.5 mg/L in C19, and free SO_2_ was 16 ± 1.8 mg/L compared with 31 ± 2.5 mg/L (Table 2). Elevated storage temperature is known to accelerate SO_2_ decline and related ageing reactions [18,19]. Here, lower SO_2_ was observed after storage at measured in-bag temperatures of 25–34 °C rather than 50 °C. Actual post-transport shelf life was not measured.

No significant between-condition difference was reported for pH, ethanol content, or titratable acidity. Total phenols, expressed as catechins, also did not differ significantly (1.02 ± 0.12 g/L in C19 against 1.10 ± 0.07 g/L in C50) (Table 2).

### 3.2. Physical Parameters

**Figure 3:**
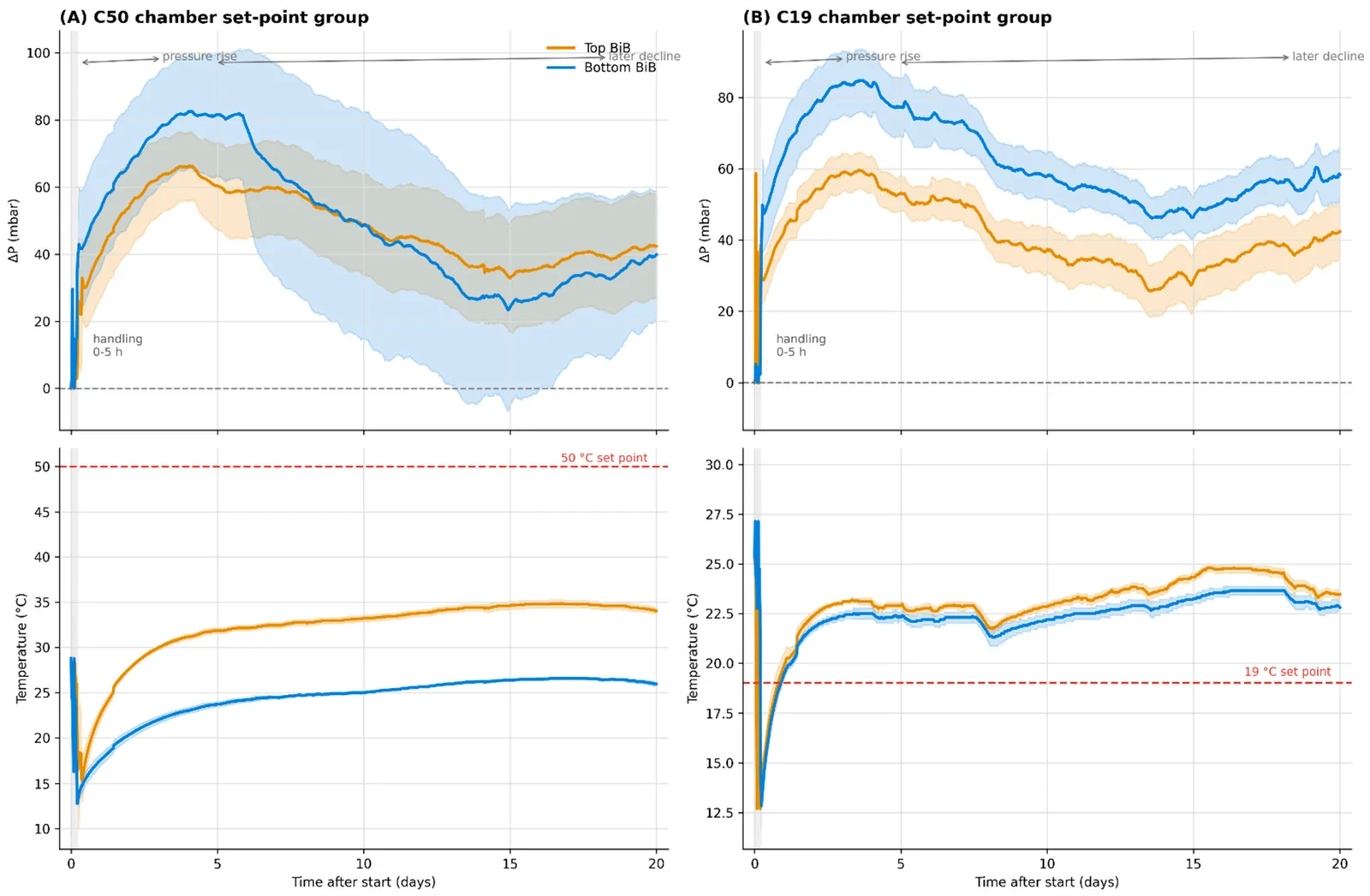
Main-trial in-bag pressure and temperature over 20 days. (**Upper panels**) show pressure change relative to each sensor’s initial valid pressure (ΔP); (**lower panels**) show measured in-bag temperature. (**A**) C50 set-point chamber; (**B**) C19 set-point chamber. Solid lines are means and shaded bands are ±1 SD. Usable sensor traces were *n* = 3 per position except bottom C19, for which *n* = 2. Dashed red horizontal lines in the temperature panels mark chamber set points; dashed horizontal lines in the pressure panels mark ΔP = 0. The left shaded band marks the first 5 h handling/stacking interval, which is expanded in Figure 4 and excluded from the primary pressure analysis.

Three stacks of 3 L BiB units were monitored for 20 days in each set-point chamber (Figure 3; Table 1). Baseline-referred pressure showed a short early transient, followed by a broad rise-and-fall pattern. The traces also shared substantial common-mode behaviour, so the absolute pressure trajectory cannot be attributed only to package pressurisation and relaxation. Individual post-handling pressure maxima occurred between day 3 + 13 h and day 5 + 19 h after the start of monitoring.

**Figure 4:**
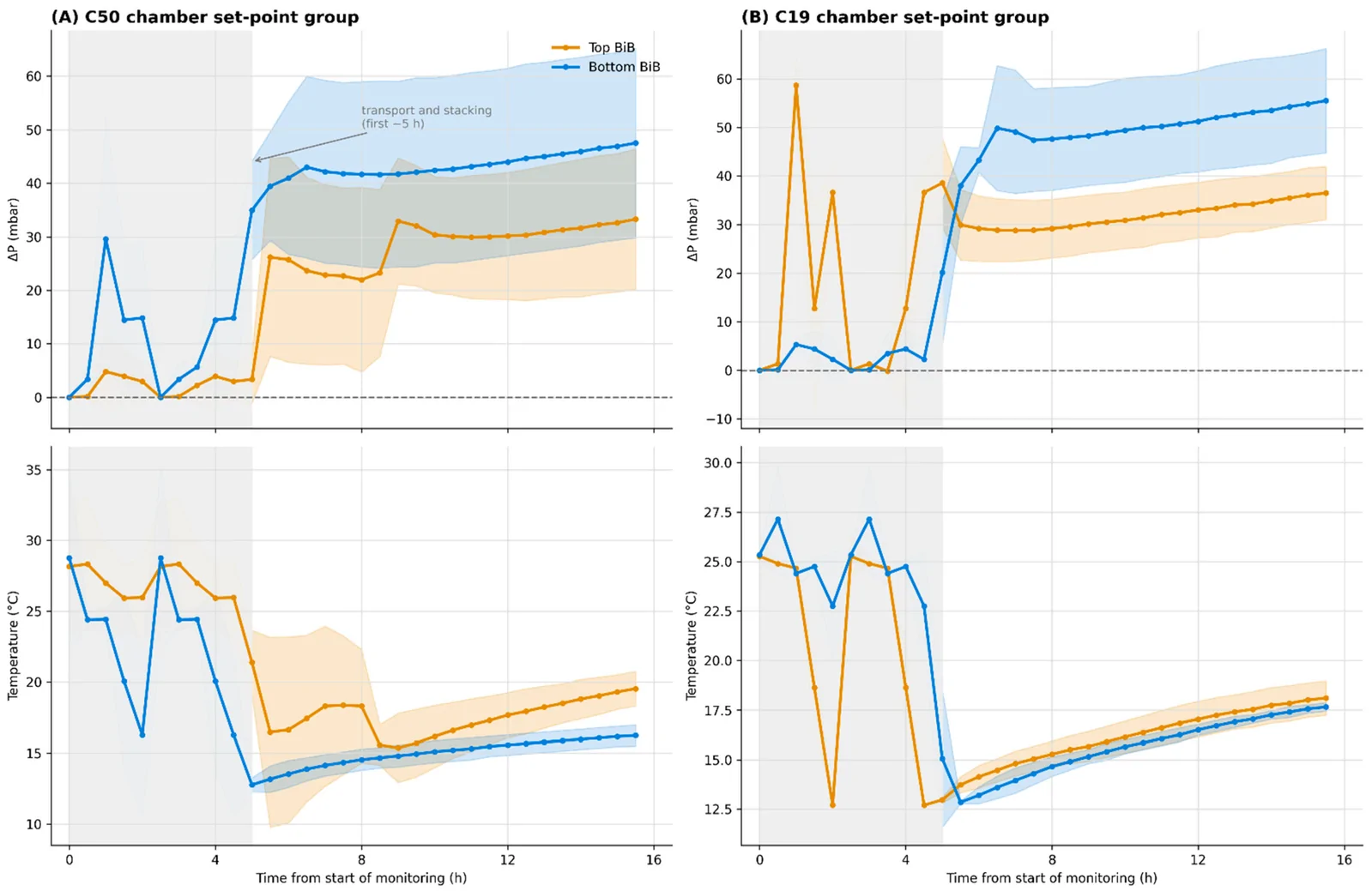
First 16 h of the main-trial record. The shaded interval marks the first 5 h handling/stacking period. (**Upper panels**) show ΔP and (**lower panels**) show measured in-bag temperature for C50 (**A**) and C19 (**B**). Solid lines are means, markers are 30 min readings, and shaded bands are ±1 SD; amber denotes top and blue denotes bottom BiBs.

The first 16 h are shown separately in Figure 4, with the first 5 h marked as the handling/stacking interval. That interval was not a controlled storage exposure, and the 30 min sampling interval was too coarse to resolve rapid transients. In a sensitivity analysis, including the first 5 h did not change the primary peak-pressure endpoint: the full-record maximum ΔP matched the post-handling maximum for all 11 usable sensors. The interval is therefore reported descriptively but excluded from the primary storage analysis.

**Figure 5:**
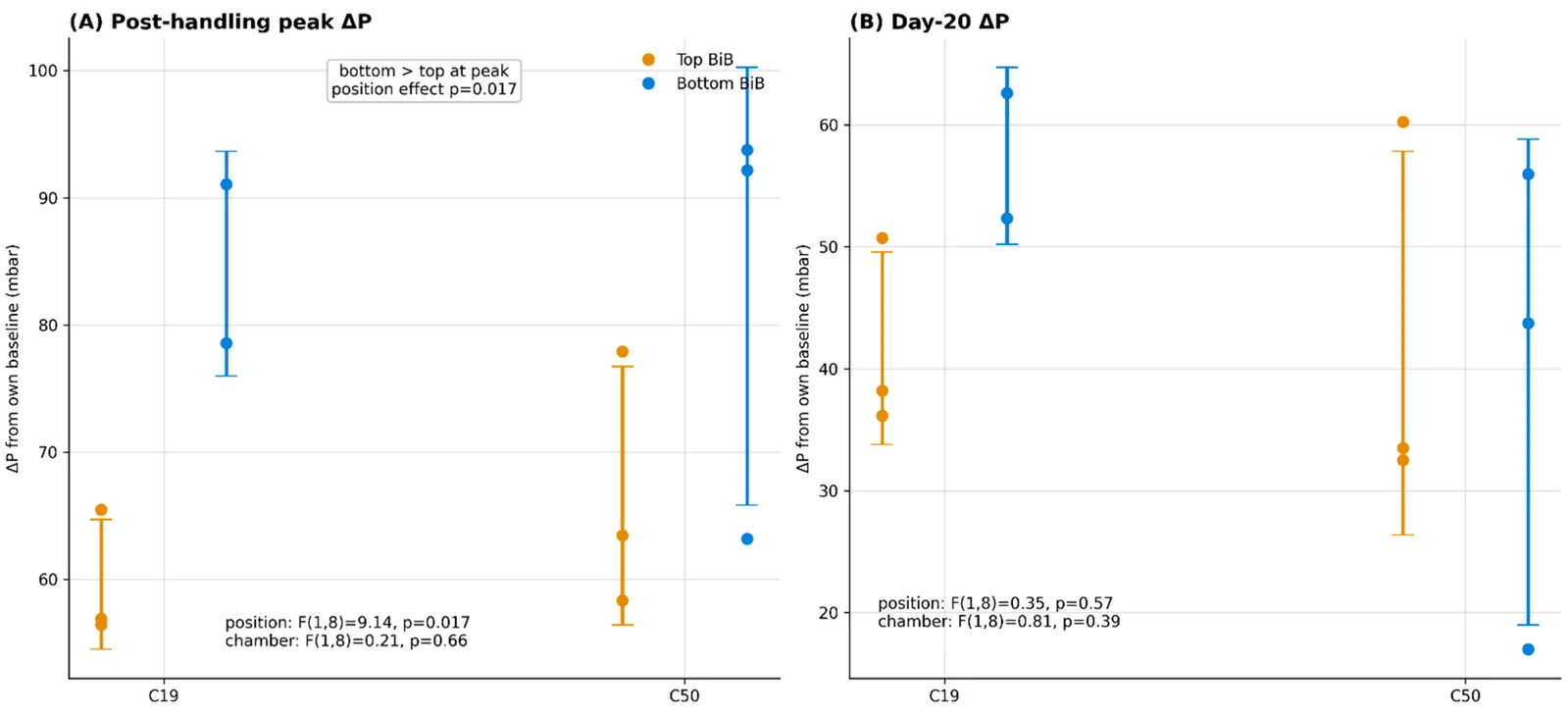
Sensor-level ΔP summaries by stack position and set-point group. (**A**) Post-handling peak; peaks occurred between day 3 + 13 h and day 5 + 19 h after start. (**B**) Residual value on day 20. Circles are individual sensors and bars represent mean ± 1 SD; usable traces were *n* = 3 per position except bottom C19 (*n* = 2). Uncorrected peak ΔP was higher in bottom than top units in exploratory analysis (F(1,8) = 9.14; *p* = 0.017). Common-mode diagnostics showed strong shared absolute-pressure structure, but matched-time residual comparison retained position term. Result is therefore interpreted as exploratory because of small unbalanced sensor-level design, missing bottom C19 trace, absence of barometric reference logging, and lack of retained stack pairings (Section 3.2).

After the handling window, uncorrected peak ΔP ranged from about 56 to 94 mbar. In C50, mean peak ΔP was 83.0 ± 17.2 mbar in the bottom units and 66.6 ± 10.1 mbar in the top units. In C19, the corresponding values were 84.8 ± 8.8 and 59.6 ± 5.1 mbar (Figure 5A). In the exploratory sensor-level Type II ANOVA, stack position was associated with uncorrected peak ΔP (F(1,8) = 9.14; *p* = 0.017), with an adjusted bottom-minus-top difference of 20.4 mbar (95% CI 4.8–35.9 mbar). The set-point factor was not statistically detectable (F(1,8) = 0.21; *p* = 0.66), and the interaction was not significant (*p* = 0.56). By day 20, neither position (F(1,8) = 0.35; *p* = 0.57) nor set-point factor (F(1,8) = 0.81; *p* = 0.39) was statistically detectable (Figure 5B).

The measured in-bag temperature differed markedly from the set points (Figure 3). In C19, late temperatures averaged 23.5 ± 0.2 °C at the top and 22.8 ± 0.3 °C at the bottom. In C50, packages remained far below the set point, with 34.2 ± 0.4 °C at the top and 26.1 ± 0.2 °C at the bottom. The warmest individual main-trial sensor reached 35.3 °C. By day 20, temperatures were stable and far below 50 °C, indicating that the effective thermal environment around the main-trial C50 packages did not reach the programmed set point; this is consistent with sustained under-delivery, stratification, or loading-related heat-transfer limitation rather than only a short thermal lag. The pressure comparison must therefore be read with the local temperature gradient in mind. The position-related peak-pressure offset was not aligned with the measured thermal gradient. In C19, the top–bottom temperature difference was small, about 0.7 °C, while the bottom-minus-top mean peak ΔP was about 25.2 mbar. In C50, the thermal gradient was much larger, about 8.1 °C, but the bottom-minus-top mean peak ΔP was smaller, about 16.4 mbar. This pattern argues against a simple temperature-drift or thermal-expansion explanation for the peak-pressure offset. It is more consistent with a position-/load-related package response under the tested stacking configuration.

Internal diagnostics showed strong common-mode structure across pressure traces: leave-one-out correlations between each sensor and the mean of the remaining sensors averaged 0.94. This indicates that the absolute pressure trajectories shared a substantial common-mode component, consistent with external or chamber-level pressure variation superimposed on package-level responses. However, a common additive pressure component does not by itself explain a top-versus-bottom contrast when sensors are compared at matched endpoint times. When common-mode residuals were evaluated at the original uncorrected peak times, the position term remained statistically detectable (F(1,8) = 9.02; *p* = 0.017). So, the alternative maximum-residual endpoint was not statistically detectable (F(1,8) = 3.21; *p* = 0.111). However, this represents a different endpoint definition rather than a direct correction of the original peak-pressure endpoint. Leave-one-out analysis of the uncorrected endpoint gave position *p*-values from 0.0007 to 0.051. The pressure result is therefore interpreted as a transient position-related offset superimposed on strong common-mode absolute-pressure variation, and it remains exploratory because of the small unbalanced sensor-level design, the missing bottom C19 trace, the absence of barometric reference logging, and the lack of retained stack pairings. These diagnostics are summarised visually in Supplementary Figure S1.

### 3.3. Verification at the C50 Set Point

The verification trial used one three-box stack at the C50 set point to document package behaviour closer to the intended high-temperature exposure. Four sensors were installed: V26 and V62 in the uppermost BiB and V27 and V64 in the lowermost BiB. Pressure and temperature were logged every minute for 15 days. V26 stopped after about 4 days 8 h; the remaining three sensors completed the record. The trial provides supporting observations for this configuration only.

**Figure 6:**
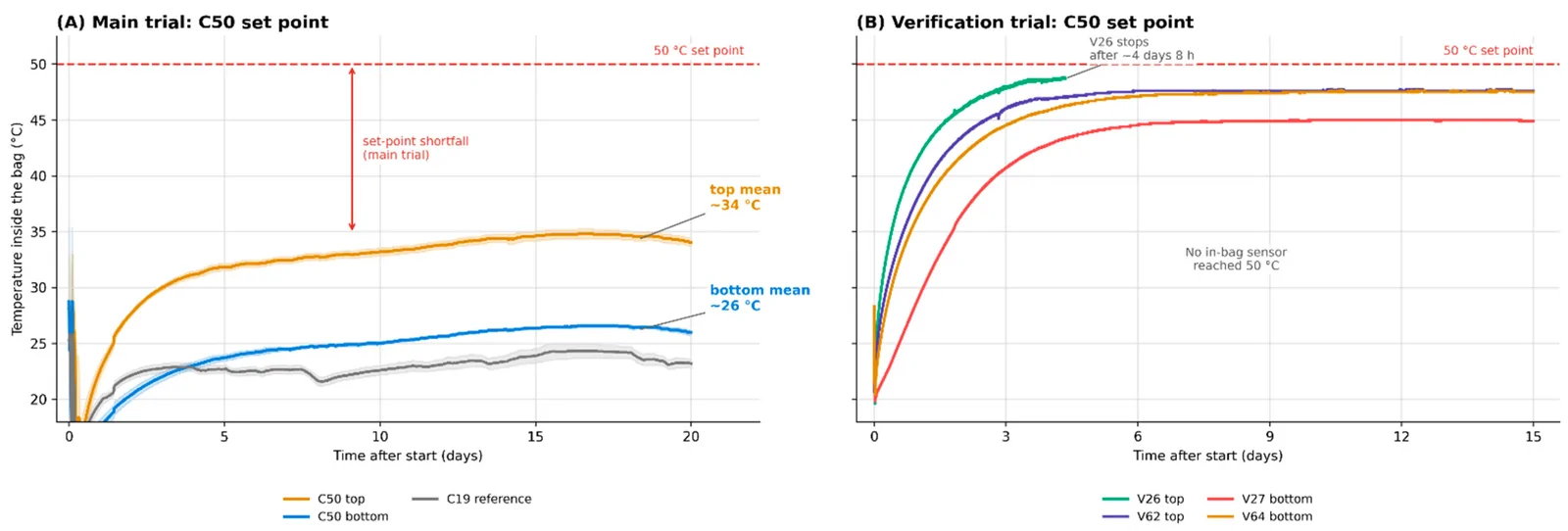
In-bag temperature in two C50 set-point trials. (**A**) Main trial, 20 days, with C19 reference shown in grey. (**B**) Verification trial, 15 days. Solid lines in panel (**A**) are means with ± 1 SD; panel (**B**) shows individual sensors. Dashed horizontal line marks 50 °C chamber set point. No in-bag sensor reached 50 °C.

**Figure 7:**
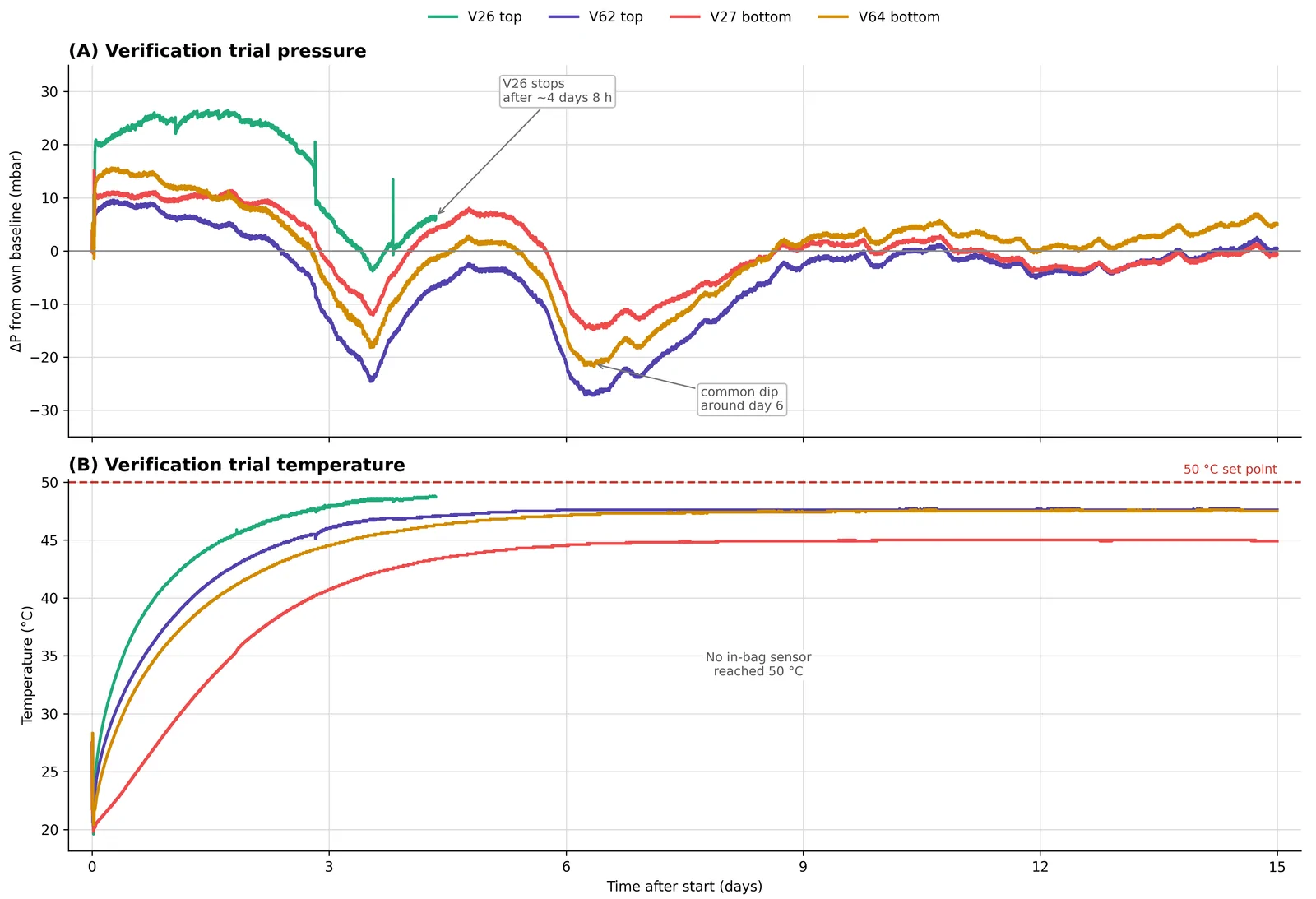
Verification trial ΔP (**A**) and measured in-bag temperature (**B**) over 15 days in one three-box stack at C50 set point. Sensors are shown individually by position; V26 stopped after about 4 days 8 h. Pressure peaks ranged from 9.6 to 26.5 mbar. No in-bag sensor reached 50 °C. Common dip around day 6 indicates shared pressure component; no barometric reference was logged.

Verification temperatures approached the chamber set point more closely than in the main trial but did not reach 50 °C (Figure 6B and Figure 7B). Maximum temperatures were 48.8 °C for V26, 47.7 °C for V62, 45.0 °C for V27, and 47.6 °C for V64. From days 14–15, V62 averaged 47.6 °C, and the two bottom sensors averaged about 46.3 °C. These values were about 13.4 °C warmer at the top and 20.2 °C warmer at the bottom than late C50 temperatures in the main trial. The difference may reflect chamber performance, loading, airflow, scale, geometry, or sensor location, but the records do not identify a single cause.

Verification ΔP changes were small and transient (Figure 7A). Relative to each sensor’s first valid pressure, early peaks ranged from 9.6 to 26.5 mbar. The traces then declined, shared a negative excursion around day 6, and returned towards baseline, while in-bag temperatures were about 45–48 °C. The common movement again indicates that the pressure change trajectory includes components other than package pressure alone. The one-stack design limits this trial to supporting evidence for the tested configuration.

### 3.4. Limitations and Future Perspectives

This preliminary study used commercial white wine and the 3 L BiB format. Grape variety and vintage information was unavailable. Chemical analyses were grouped as C19/C50 and were not position-resolved, so they cannot be linked directly to top/bottom pressure behaviour. Raw chemical replicate values were not retained in the available study records. No microbiological measurements, viable cell counts, sensory analysis, or post-transport storage follow-up were performed. Malolactic or other microbial contributions therefore cannot be excluded, and the implications of lower SO_2_ reserves for shelf life remain untested.

The main design limitation is the mismatch between the chamber set point and in-bag temperature. In C50, late in-bag temperatures were 34.2 ± 0.4 °C at the top sensors and 26.1 ± 0.2 °C at the bottom sensors. This was not a 20-day wine exposure to 50 °C. Chamber model, volume, fan power, airflow, chamber air temperature, and pallet occupancy were not recorded, preventing the identification of the specific cause of the effective thermal environment failing to reach the programmed C50 set point. There was one chamber per set point, so chamber identity and set point are confounded.

The pressure analysis used 11 sensor traces, with one missing bottom sensor and one *n* = 2 cell. Stack IDs and sensor-to-stack pairings were not retained, so paired stack-level analysis was not possible. No barometric reference sensor was logged. Strong common-mode correlations among pressure traces limit the interpretation of the shared absolute-pressure trajectory, but they do not by themselves explain the top-versus-bottom contrast at matched endpoint times. The pressure-position result remains exploratory because the analysis was based on 11 sensor traces, as one bottom C19 trace was missing, no barometric reference sensor was logged, and stack identifiers or sensor-to-stack pairings were not retained. Baseline referencing reduces static offsets. It does not remove temperature-dependent drift, ambient barometric variation, or possible effects from the oil-filled casing and displaced headspace. No bench calibration in the wine matrix was available. The first 5 h were excluded from the primary storage analysis; including them did not change the peak endpoint. The verification trial used one stack, one sensor stopped after about 4 days 8 h, and no sensor reached 50 °C. Sensor deployment is discussed only as sentinel, validation, or quality-control monitoring because no techno-economic analysis was performed. For future pallet-scale validation of 3 L BiB packaging, sensor density should be treated as a design parameter rather than assumed from this preliminary trial. As a practical starting point, at least six in-bag pressure sensors per pallet-scale configuration would be desirable, with one top-position and one bottom-position BiB instrumented in at least three stacks or pallet locations. When resources allow, 8–12 sensors distributed across stack height and pallet position would give better coverage of the local load and thermal heterogeneity. These numbers should be considered preliminary, because no formal deployment density or techno-economic optimisation was performed in this study. Before implementation, sensors should be checked against a pressure reference, tested for thermal drift over the expected storage temperature range, verified for sealing after insertion, and supported by independent barometric and air temperature logging.

Despite these limitations, the study shows why direct package-level measurement matters. Chamber set points did not predict realised wine temperature, and sensor-level pressure summaries suggested position-related offsets under the tested stacking configuration. Future work should combine replicated chambers with chamber air and barometric logging, bench calibration, position-resolved chemistry, microbiology, realistic fluctuating temperature and vibration profiles, broader package formats, and cost–benefit evaluations of sentinel sensing.

## 4. Conclusions

This study monitored pressure and temperature inside palletised 3 L BiB wine units under static chamber conditions. The set-point labels C19 and C50 did not represent the temperature experienced by the wine. In the main trial, C50 packages stabilised at 34.2 ± 0.4 °C at the top and 26.1 ± 0.2 °C at the bottom, while C19 packages were about 23 °C. The main trial therefore supports conclusions about the realised set-point groups and measured package temperatures, not about a 20-day wine exposure to 50 °C.

Bottom-position BiBs had a larger mean uncorrected peak ΔP than top-position BiBs under the tested stacking configuration (adjusted difference of 20.4 mbar; F(1,8) = 9.14; *p* = 0.017). Individual post-handling pressure maxima were reached between day 3 + 13 h and day 5 + 19 h after the start of monitoring, well after the initial 5 h handling/stacking interval. Internal diagnostics showed a strong common-mode structure across the absolute pressure traces. However, when common-mode residuals were evaluated at the original uncorrected peak times, the position term remained statistically detectable (F(1,8) = 9.02; *p* = 0.017). The alternative maximum-residual endpoint was not statistically detectable (F(1,8) = 3.21; *p* = 0.111). However, this represents a different endpoint definition rather than a direct correction of the original peak-pressure endpoint. The pressure result is therefore interpreted as a transient position-related offset superimposed on common-mode absolute-pressure variation, and it remains exploratory because of the small unbalanced sensor-level design, the missing bottom C19 trace, the absence of barometric reference logging, and the lack of retained stack pairings. Neither factor was statistically detectable by day 20. Including the first 5 h did not change the uncorrected peak endpoint. Wine from C50 showed lower free and total SO_2_, higher volatile acidity, and a numerically higher lactic acid value than wine from C19, while pH, ethanol, titratable acidity, and total phenols did not differ significantly. These are bulk chamber-group chemistry results; they were not resolved by package position. Shelf life, sensory quality, and microbiology after storage were not tested. In the verification trial, one stack reached 45.0–48.8 °C across sensors but still did not reach 50 °C, and pressure changes were small and transient. The practical implication is to use selected sentinel packages or validation deployments to measure actual in-package conditions, with deployment density and economic feasibility left for future work.

## Figures and Tables

**Table 1:** Structure of experimental samples and placement of pressure sensors.

Trial	Set-Point Label	Duration	Stack	Boxes in Stack (Height)	MEMS Sensors
Main	C19 (19 °C)	20 days	Stack 1	4 (128 cm)	Top + bottom BiB
Main	C19 (19 °C)	20 days	Stack 2	3 (97 cm)	Top + bottom BiB
Main	C19 (19 °C)	20 days	Stack 3	3 (97 cm)	Top + bottom BiB *
Main	C50 (50 °C)	20 days	Stack 1	4 (128 cm)	Top + bottom BiB
Main	C50 (50 °C)	20 days	Stack 2	3 (97 cm)	Top + bottom BiB
Main	C50 (50 °C)	20 days	Stack 3	3 (97 cm)	Top + bottom BiB
Verification	C50 (50 °C)	15 days	Stack 1	3 (97 cm)	2 top + 2 bottom BiB

Main trial: This used twelve MEMS pressure sensors in total—one in the top and one in the bottom BiB of each of three stacks in each set-point chamber. C19 and C50 were applied in parallel in separate climate-controlled chambers. * The bottom sensor of one C19 stack did not return a complete record, leaving eleven usable traces for pressure analysis.

**Table 2:** Chemical parameters after 20 days in C19 and C50 set-point chambers. C19 and C50 identify chamber set points, not measured in-bag wine temperatures. Values are mean ± standard deviation (*n* = 3).

Sample	Lactic Acid (g/L)	Malic Acid (g/L)	pH	Titratable Acidity (g/L of Tartaric Acid)	Volatile Acidity (g/L of Acetic Acid)	Total Phenols (g/L of Catechins)	Ethanol Content (%*v*/*v*)	Total Sulphur Dioxide Content (mg/L)	Free Sulphur Dioxide Content (mg/L)
C19 chamber set point	0.16 ± 0.05 ^B^	1.1 ± 0.23 ^A^	3.4 ± 0.1 ^A^	6.6 ± 0.04 ^A^	0.30 ± 0.02 ^B^	1.02 ± 0.12 ^A^	11.6 ± 0.6 ^A^	50 ± 5.5 ^A^	31 ± 2.5 ^A^
C50 chamber set point	0.29 ± 0.02 ^A^	1.0 ± 0.16 ^A^	3.4 ± 0.1 ^A^	6.6 ± 0.13 ^A^	0.39 ± 0.03 ^A^	1.10 ± 0.07 ^A^	11.6 ± 0.9 ^A^	25 ± 8.2 ^B^	16 ± 1.8 ^B^

Different superscript uppercase letters (A–B) indicate statistically significant differences between samples within each column (*p* ≤ 0.05).

## Data Availability

The original contributions presented in this study are included in the article and Supplementary Materials. Further inquiries can be directed to the corresponding author.

## Supplementary Materials

Figure S1 is available on Zenodo: https://doi.org/10.5281/zenodo.21921020. Supplementary Figure S1. Common-mode diagnostic for the main-trial pressure endpoint. Figure S1 includes trace-wise common-mode correlations, a matched-time residual comparison, and leave-one-out sensitivity analysis.
